# Genome-wide identification and functional analysis of *Cellulose synthase* gene superfamily in *Fragaria vesca*

**DOI:** 10.3389/fpls.2022.1044029

**Published:** 2022-11-03

**Authors:** Hexin Huang, Shuai Zhao, Junli Chen, Tianxiang Li, Ganggang Guo, Ming Xu, Sufeng Liao, Ruoting Wang, Jiayi Lan, Yangxin Su, Xiong Liao

**Affiliations:** ^1^ College of Horticulture, Fujian Agriculture and Forestry University, Fuzhou, China; ^2^ College of Life Science, Fujian Agriculture and Forestry University, Fuzhou, China; ^3^ College of Agriculture, Key Laboratory of Crop Biotechnology in Fujian Province University, Fujian Agriculture and Forestry University, Fuzhou, China

**Keywords:** *Fragaria vesca*, *CesA/Csl*, cell wall, fruit firmness, fruit ripening

## Abstract

The *Cellulose synthase* (*CesA*) and *Cellulose synthase-like* (*Csl*) gene superfamilies encode key enzymes involved in the synthesis of cellulose and hemicellulose, which are major components of plant cell walls, and play important roles in the regulation of fruit ripening. However, genome-wide identification and functional analysis of the *CesA* and *Csl* gene families in strawberry remain limited. In this study, eight *CesA* genes and 25 *Csl* genes were identified in the genome of diploid woodland strawberry (*Fragaria vesca*). The protein structures, evolutionary relationships, and *cis*-acting elements of the promoter for each gene were investigated. Transcriptome analysis and quantitative real-time PCR (qRT-PCR) results showed that the transcript levels of many *FveCesA* and *FveCsl* genes were significantly decreased during fruit ripening. Moreover, based on the transcriptome analysis, we found that the expression levels of many *FveCesA/Csl* genes were changed after nordihydroguaiaretic acid (NDGA) treatment. Transient overexpression of *FveCesA4* in immature strawberry fruit increased fruit firmness and reduced fresh fruit weight, thereby delaying ripening. In contrast, transient expression of *FveCesA4*-RNAi resulted in the opposite phenotypes. These findings provide fundamental information on strawberry *CesA* and *Csl* genes and may contribute to the elucidation of the molecular mechanism by which *FveCesA*/*Csl*-mediated cell wall synthesis regulates fruit ripening. In addition, these results may be useful in strawberry breeding programs focused on the development of new cultivars with increased fruit shelf-life.

## Introduction

The firmness of strawberry (*Fragaria* spp.) fruit is a crucial agronomic trait that is closely associated with its storage life and commercial value. Fruit development in strawberry is divided into two stages: the early growth stage and the ripening stage (Liao et al., 2018). During the early stage, the fruit enlarges. During the ripening stage, the fruit texture undergoes dramatic changes, which is defined as fruit softening, mainly as a result of alterations in cell wall structure and composition (Moya-León et al., 2019). The *Cellulose synthase* gene superfamily, which belongs to the glycosyltransferase-2 gene superfamily, is presumed to be involved in the synthesis of major components of the primary and secondary cell walls, such as cellulose and hemicellulose polysaccharides (Czaja et al., 2007). Hence, the mining of genes encoding cellulose synthases that function in fruit development may enable manipulation of fruit ripening and thereby enhance fruit storability.

The *Cellulose synthase* gene superfamily comprises the *Cellulose synthase A* (*CesA*) and *Cellulose synthase-like* (*Csl*) families, which can be resolved into the CesA clade and ten Csl clades, i.e., CslA-CslH, CslJ and CslM (McFarlane et al., 2014). Among the Csl clades, the CslA, CslC and CslD lineages are prevalent in all terrestrial plants (Farrokhi et al., 2006). The CslB lineages are considered to be dicotyledons-specific, whereas the CslF and CslH lineages were indicated to be restricted to grasses (Yin et al., 2009). Cellulose catalyzed by CesA plays a central role in cell morphogenesis and cell wall integrity, which predominantly determines plant development and growth (Cosgrove, 2005; Persson et al., 2007; Liu et al., 2016; Hu et al., 2018a; Hu et al., 2018b). In *Arabidopsis* (*Arabidopsis thaliana*), *AtCesA1*, *AtCesA3* and *AtCesA6-like* genes (*AtCesA2*, *AtCesA5*, *AtCesA6*, and *AtCesA9*) are essential for the synthesis of primary cell wall cellulose, whereas *AtCes4*, *AtCes7*, and *AtCes8* are responsible for the synthesis of secondary cell wall cellulose (Taylor et al., 2003; Desprez et al., 2007; Persson et al., 2007; Malinovsky et al., 2014). Overexpression of *AtCesA2*, *AtCesA5*, and *AtCesA6* in *Arabidopsis* causes enhanced cell elongation and divisiona as well as increased secondary cell wall deposition which, consequently, results in greater biomass production (Hu et al., 2018a). In maize (*Zea mays*), the *ENB1* gene encoding cellulose synthase is predominantly expressed in the basal endosperm transfer layer (BETL) of the endosperm during kernel development (Wang et al., 2022). Loss-of-function of *ENB1* causes defective cell wall ingrowths in BETL cells, which, in turn affects nutrient absorption and, ultimately, results in severe degradation of the endosperm (Wang et al., 2022).

Genes in the *Csl* family, which show significant similarity to *CesA* gene family (Richmond and Somerville, 2000; Cantarel et al., 2009), are grouped into ten subfamilies, including the *CslA-H*, *CslJ*, and *CslM* subfamilies (Richmond and Somerville, 2000; Hazen et al., 2002; Farrokhi et al., 2006; Cantarel et al., 2009; Little et al., 2018). Evidence to date indicates that diverse cell wall polysaccharides catalyzed by *Csl* gene family members are crucial for plant development and for biotic or abiotic stress tolerance. Several members of the Arabidopsis *CslA* family function in the synthesis of mannan polysaccharides (Dhugga et al., 2004; Liepman et al., 2005; Goubet et al., 2009; Liepman and Cavalier, 2012). Overexpression of *AtCslA2*, *AtCslA7*, and *AtCslA9* enhances glucomannan biosynthesis and causes defective embryogenesis, resulting in retard development and occasional embryo death (Goubet et al., 2009). The *CslD* gene family considered to be involved in the synthesis of xylan, homogalacturonan, and mannan polysaccharides (Bernal et al., 2007) and is required for the development of tip-growing cells. In *Arabidopsis*, loss-of-function of *AtCslD2* and *AtCslD3* results in a defective root-hair phenotype, and *AtCslD1* and *AtCslD4* mutants exhibit a defective pollen tube phenotype. Intriguingly, constitutive overexpression of the *Csl* gene *Soly07g043390* in tomato (*Solanum lycopersicum*) significantly enhances symptomatic tolerance and further results in improved plant growth, fruit size, and yield in the tomato-*Tomato yellow leaf curl virus*-pathosystem (Park et al., 2011; Verhertbruggen et al., 2011; Yin et al., 2011; Dhugga, 2012; Yang et al., 2020; Choe et al., 2021). Therefore, *Csl* genes may be utilized to potentially strengthen the plant immunity system and maintain crop productivity.

Together with the mining of large-scale plant genomic sequence data, the *Cellulose synthase* gene superfamily has been identified in a variety of plants, such as rice (*Oryza sativa*) (Hazen et al., 2002), maize (Appenzeller et al., 2004; Li et al., 2019), tomato (Song et al., 2019), and *Physcomitrella patens* (Roberts and Bushoven, 2007). However, genome-wide characterization of the genes associated with cellulose synthesis in strawberry remains to be elucidated. In the present study, to mine the potential cellulose synthesis genes that influence fruit ripening in strawberry, eight *CesA* and 25 *Csl* genes were identified in the genome of diploid woodland strawberry. The significantly down-regulated candidate *FveCesA* and *FveCsl* genes were screened by transcriptome analysis and qRT-PCR. In addition, transient overexpression and knock-down experiments were performed to verify the function of candidate genes involved in the regulation of fruit ripening. These results may facilitate analysis of the mechanism by which *FveCesA*/*Csl*-mediated cell wall synthesis regulates fruit ripening, and the discovery of candidate genotypes for improved fruit shelf-life in strawberry.

## Materials and methods

### Plant materials and growth conditions

Diploid woodland strawberry (*F. vesca*) seedlings were planted in plastic pots (9 cm × 9 cm) containing an equal mixture of vermiculite and peat soil. The plants were grown in a growth room under a 16 h/8 h (light/dark) photoperiod at 22°C with 60% relative humidity. Once fully open, flowers were pollinated manually every 2 days with a pollination stick.

### Identification and characterization of *Cellulose synthase* superfamily members in the strawberry genome

The *F. vesca* v4.0 genome and v4.0.a2 annotation files were downloaded from the GDR database (https://www.rosaceae.org/). The strawberry protein database was searched using the TBtools software (Chen et al., 2020). The amino acid sequences for members of the *Arabidopsis* cellulose synthase family (10 *AtCesA* and 30 *AtCsl* genes) were extracted from the TAIR database (https://www.arabidopsis.org/) and were employed as queries for BLAST searches with a cutoff *E-*value of 1e^-5^ in the strawberry protein database. A total of eight *FveCesA* and 25 *FveCsls* genes were identified by a bi-directional BLAST analysis using the TBtools software and the NCBI Blastp tools. The NCBI Conserved Domain Database (http://www.ncbi.nlm.nih.gov/cdd) and Pfam database (http://pfam.xfam.org/) were used to predict the conserved domains in all putative FveCesA and FveCsl proteins. The TBtools software was used to predict the protein molecular weight, isoelectric point, and gene structure. The Plant-mPLoc server (http://www.csbio.sjtu.edu.cn/bioinf/plant-multi/) was used to predict the subcellular localization of the putative proteins.

### Phylogenetic relationship, chromosomal location, and synteny analysis

Multiple sequence alignment of the cellulose synthase protein sequences *F. vesca*, *A. thaliana*, and *O. sativa* was performed with MUSCLE software. Maximum likelihood phylogenetic trees were constructed, with topological support assessed with 1000 bootstrap replicates, under the LG+G+I+F substitution model using MEGA7 (Kumar et al., 2016). The chromosomal distribution of the identified *FveCesA* and *FveCsl* genes, and the syntenic relationships between CesA/Csl homologs of *F. vesca* and *Arabidopsis* were analyzed and visualized using TBtools.

### Gene structure, conserved domain, and motif composition analysis

The coding sequences (CDSs) and corresponding genome sequences of the *FveCesA* and *FveCsl* genes were extracted from the *F. vesca* genome and used to predict the exon-intron structures. The conserved domains and motifs of FveCesAs and FveCsl proteins were investigated using the NCBI Conserved Domain Database (https://www.ncbi.nlm.nih.gov/Structure/cdd/wrpsb.cgi) and the MEME Suite tools (https://meme-suite.org/meme/), respectively. Aschematic diagram of gene structure, conserved domains, and motif composition was generated and re-edited with TBtools.

### Promoter *Cis*-acting element distribution analysis

The 2000 -bp sequences upstream of the start codon of each *FveCesA* and *FveCsl* gene was extracted as the promoter region and submitted to the PlantCARE database (https://bioinformatics.psb.ugent.be/webtools/plantcare/html/) for prediction of the *cis*-acting elements. A heat map of the *cis*-acting elements of each *FveCesA* and *FveCsl* gene was visualized using TBtools.

### Transcriptome data analysis

For transcriptome analysis, strawberry fruits at the small white stage [about 17 days after pollination (DAP)], big white stage (about 21 DAP), pre-turning stage (white receptacle with red achenes), pink stage, and red stage (two or three days after the pink stage) were collected for RNAseq analysis. To inhibit endogenous ABA synthesis, the fruits at big green stage (12-14 DAP) were treated with NDGA and harvested five days after treatment. To investigate the expression patterns of *FveCesA* and *FveCsl* genes during fruit development, the raw data for the above diploid woodland strawberry fruit transcriptome (PRJNA522346) at different developmental stages (Gu et al., 2019) were downloaded from the NCBI database using Aspera software. The Bam files were generated by aligning the transcriptome reads to the *F. vesca* genome using HISAT2 software. Read counts aligned to each *FveCesA* and *FveCsl* gene were counted using the featureCounts program. The DESeq2 R package was used to standardize read counts to obtain transcripts per kilobase million (TPM) values. A heat map of the gene expression patterns was visualized using the TBtools.

### RNA isolation and qRT- PCR analyses

To verify the expression pattern of *FveCesA*/*Csl* genes during fruit ripening, according to previous reports, the fruit development of “Yellow Wonder” was divided into 12 stages from fully open flower, including S1 (open flower), S2 (2-4 DAP), S3 (5-7 DAP), S4 (8-10 DAP), S5 (11-13 DAP), S6 (14-16 DAP), S7 (17-19 DAP), RS1 (20-22 DAP), RS2 (23-25 DAP), RS3 (26-28 DAP), RS4 (29-31 DAP), and RS5 (32-34 DAP) (Liao et al., 2018). Total RNA of strawberry fruits in the early development stage (S1, S3 and S5) and the ripening stage (RS4) were isolated using the polysaccharide and polyphenolics-rich RNAprep Pure Kit (Tiangen) in accordance with the manufacturer’s instructions. The cDNA was synthesized from total RNA using the HiScript II Q RT SuperMix for qPCR reagent Kit (Vazyme). The RT-qPCR assay was performed on a CFX96 Real-Time PCR system (Bio-Rad) using the AceQ qPCR SYBR Green Master Mix (Vazyme). *FveACTIN* was used as the internal control for normalization. The primers used for qRT-PCR analyses are listed in **Supplementary Table S1**.

### Construction of plasmid DNA

To generate an overexpression vector, the full-length CDS of *FveCesA4* was cloned into the pCAMBIA1305 vector and designated *35S::FveCesA4*. For strawberry fruit RNAi interference (RNAi) analysis, the pTRV1/pTRV2 viral-induced gene silencing (VIGS) system was employed. A 375 -bp CDS fragment (from 152 bp to 527 bp) of *FveCesA4* was amplified and inserted into the pTRV2 vector, and the construct was designated *pTRV2-FveCesA4*. The primers used in vector construction are listed in **Supplementary Table S1**.

### Transient transformation analysis in strawberry fruit

Strawberry fruits were infiltrated with *Agrobacterium* as previously described (Liao et al., 2018). For the RNAi experiment, fruits at the S4 stage were injected with Agrobacterium *tumefaciens* strain GV3101 harboring *pTRV1* in combination with the vector containing either *pTRV2* or *pTRV2-FveCesA4*. For transient overexpression, fruits at the S7 stage were injected with *A. tumefaciens* strain GV3101 harboring *35S::FveCesA4*. Approximately 15 fruits from five individual plants were selected for transient infiltration in each experiment. The fruit phenotype was evaluated 7 days after injection.

### Firmness and fruit size analysis

Fruit size was measured with a vernier calipers. Fresh fruit firmness was detected using a texture analyzer (GY-4; Handpi). Each fruit was measured five times in the equatorial region.

## Results

### Genomic identification and phylogenetic analysis of *Cellulose synthesis* gene in strawberry

To identify candidate cellulose synthesis genes, a bidirectional BLAST search was performed against the *F. vesca* reference genome using the CesA/Csl protein sequences of *Arabidopsis*. Eight *FveCesA* and 25 *FveCsl* genes were obtained. The candidate *CesA* and *Csl* genes were named consistent with their grouping in a phylogenetic analysis (**Supplementary Table S2**). Overall, the candidate proteins were predicted to range from 518 (*FveCslA1*) to 1138 (*FveCSLD1*) amino acids and from 5.93 KD (*FveCslA1*) to 12.6 KD (*FveCslD1*) in molecular weight. The pI ranged from 6.05 (*FveCslD1*) to 9.19 (*FveCslA1*). The candidate cellulose synthases were predicted to be localized in the cell membrane, chloroplasts and the Golgi apparatus (**Supplementary Table S2**).

**Figure 1:**
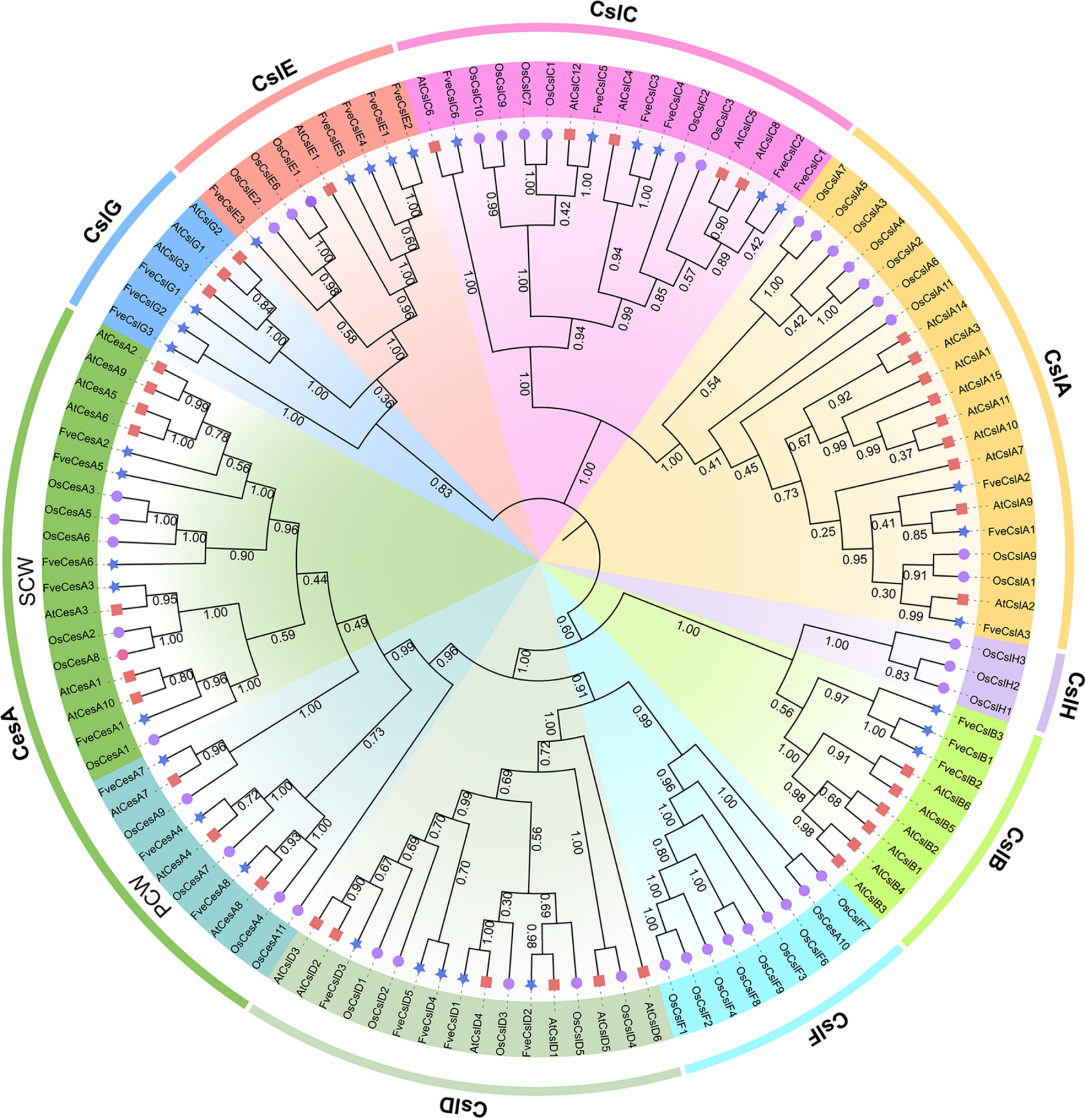
Phylogenetic relationships among the *Cellulose synthase* genes from *Arabidopsis* (red square), rice (purple circular) and strawberry (blue star). The selected CesA/Csl proteins are clustered into one CesA subfamily and nine Csl subfamilies and distinguished by different colors.

To explore the evolutionary relationships of FveCesA/Csl proteins in strawberry, 117 CesA and Csl homologs from *F. vesca*, *Arabidopsis*, and rice were selected for phylogenetic analysis (**Figure 1** and **Supplementary Table S3**). The cellulose synthase homologs of the three species were grouped into one CesA clade and eight Csl clades (designated CslA-CslH). Eight FveCesA/Csl proteins were grouped in the CesA clade, which constituted the largest clade. With regard to the remaining homologs from strawberry, three FveCesA/Csl proteins were placed in each of the CslA, CslB, and CslG clades, six were grouped in the CslC clade, and five in each of the CslD and CslE clades. No rice CesA/Csl homologs were grouped in the CslB and CslG clades. In contrast, no CesA/Csl homologs of *Arabidopsis* and strawberry were placed in the CslF and CslH clades, which were only present in rice. In addition, CslD and CesA clades were phylogenetically close, suggesting that the CesA/Csl homologs in these two lineages shared a common ancestor.

### Chromosomal distribution and synteny analysis of Cellulose synthesis genes in strawberry

**Figure 2:**
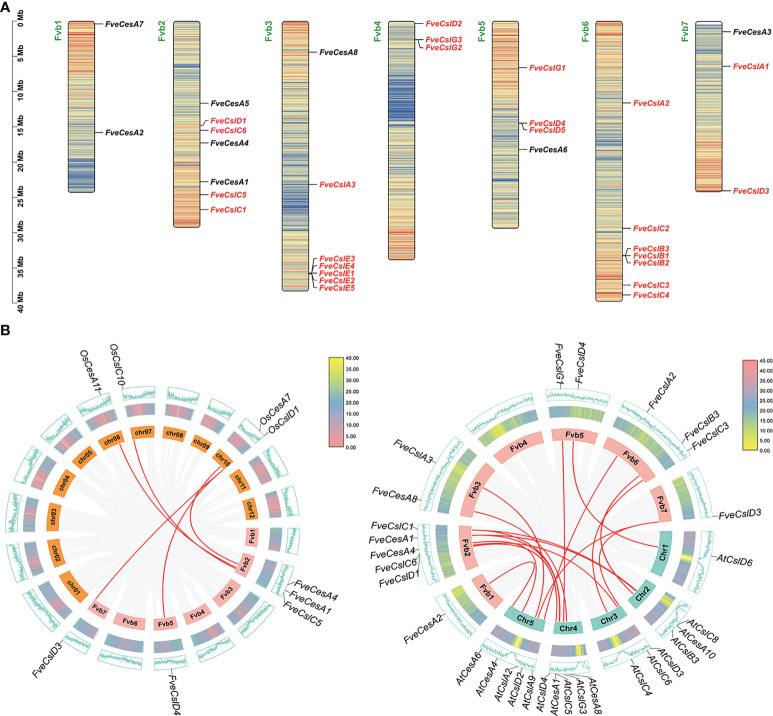
Chromosome distribution and synteny analysis of *Cellulose synthase* gene superfamily in strawberry. **(A)** Distribution diagram of *Cellulose synthase* genes in strawberry chromosome. The strawberry chromosomes are denoted as “Fvb”, strawberry *CesA* family and *Csl* families are distinguished by black and red letters, respectively; **(B)** Synteny analysis of *Cellulose synthase* genes between strawberry, *Arabidopsis* and rice. Left diagram: analysis between rice and strawberry; right diagram: analysis between *Arabidopsis* and strawberry.

The 33 cellulose synthesis genes were unequally distributed on all seven chromosomes of *F. vesca* (**Figure 2A**). The *FveCesA* genes were located on all chromosomes except Fvb4. By comparison, *FveCsl* genes were present on all chromosomes except Fvb1. Intriguingly, *FveCslB* and *FveCslE* members exhibited tandem or segmental duplication, and were located at the ends of chromosomes Fvb6 and Fvb3, respectively.

To further explore the evolutionary mechanism of the *FveCesA/Csl* gene family, comparative syntenic maps of *F. vesca* and two representative species (*Arabidopsis thaliana* and *Oryza sativa*) were constructed at the genome-wide scale. Fourteen *FveCesA*/*Csl* genes and five *FveCesA/Csl* genes exhibited collinearity relationships with those of *Arabidopsis* and rice, respectively (**Figure 2B**). Interestingly, *FveCesA1*, *FveCesA4*, *FveCslD3*, and *FveCslD4* shared collinear relationships with both of these species, suggesting that these orthologous pairs may have predated the monocotyledon-eudicotyledon divergence.

### Sequence analysis of Cellulose synthesis genes in strawberry

**Figure 3:**
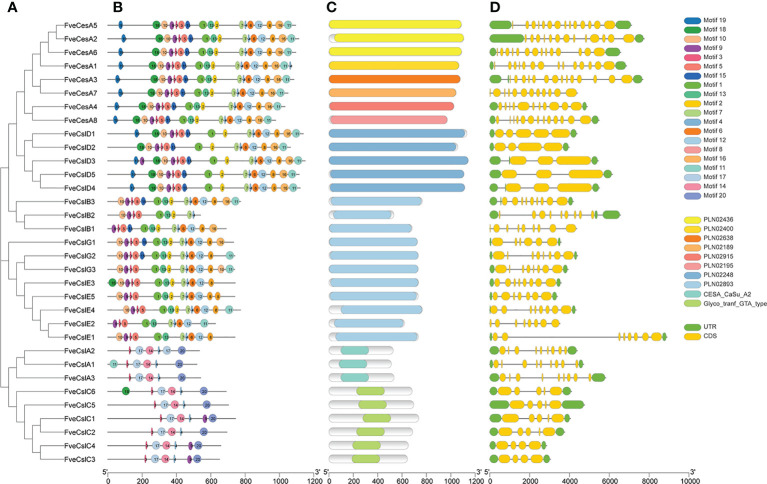
Structure characteristic of cellulose synthase gene in strawberry. **(A)** Phylogenetic analysis of FveCesA/Csl proteins. **(B)** Conserved motifs of FveCesA/Csl proteins. **(C)** Conserved domains of FveCesA/Csl proteins. **(D)** Exon-intron structures of *FveCesA/Csl* genes. UTRs are highlighted in green rectangles; exons are highlighted in yellow rectangles; introns are represented by solid lines. The scale bars indicate the length of the corresponding proteins and genes.

To clarify the structural characteristics of the *FveCesA*/*Csl* gene family, the conserved domain, motif composition, and gene structure were analyzed. Two conserved domains in FveCesA/Csl proteins were identified (**Figure 3** **)**. The 24 proteins of the CesA, CslD, CslB, CslE, and CslG clades contained the cellulose synthase conserved domain (PLN02436, PLN02400, PLN02638, PLN02189, PLN02915, PLN02248 and PLN02893). In comparison, the nine proteins of the CslA and CslC clades contained the glycosyl transferase family 2 domain (CESA_CaSu_A2 and Clyco_tranf_GTA_type) (**Figure 3B** and **Supplementary Table S4**). Consistent with the conserved domain analysis, most proteins in the CslD clade shared a similar motif composition with the CesA clade, except for the absent of motif13. Except in rare cases, proteins in the CslB, CslG, and CslE clades, containing the conserved domain PLN02893, shared similar motif orders. In contrast, the CslA and CslC clades contained a similar motif composition, but were distinguished from those of the other clades (**Figure 3A**). The majority of proteins containing similar motif compositions were grouped in the same clade and may have similar functions.

Analysis of the exon-intron organization of the *FveCesA*/*Csl* genes revealed diverse genetic structures. The number of exons in ranged from four to 14. Among the 33 *FveCesA*/*Csl* genes, the majority (66%) were spliced with more than six exons and five introns (**Figure 3C**). *FveCesA*/*Csl* genes with similar gene structures were classified together, for example, the CslD and CslC clades harbored five exons and four introns. The number of exons of the CesA clade ranged from 11 to 14, whereas the other clades contained nine (CslB clade), six to eight (CslG clade), eight (CslE clade), and nine (CslA clade). The diversity of gene structure indicated that *FveCesA/Csl* clades had experienced diverse evolutionary scenarios.

To further analyze the conserved domain of the FveCesA/Csl proteins, the amino acid sequence alignment of proteins in each clade was performed (**Supplementary Figures S1**–**S4** and **Supplementary Table S4**). We found that *Cellulose synthase* domain (cellulose_synt) was ubiquitously present in CesA, CslB, CslD, CslE, CslG subfamily proteins (**Supplementary Figures S1**, **S2**, **S4**). However, CslA and CslC subfamily proteins contained glycosyltransferase family domain (Glyco_trans_2_3), which further confirmed that these two subfamilies may have different evolutionary origination (**Supplementary Figure S4**). Almost all FveCesA/Csl proteins typically contained “D, D, D and QxxRW” active site, suggesting that they have conserved catalytic function. The “Q” amino acid of the “QxxRW” motif was replaced by other amino acids, implying that they may have functional differentiation. Unlike other families, the N- terminals of A and D contain zinc finger structures (**Supplementary Figures S1**), including conserved cysteine residues, which presumably may contribute to homologous or heteropolymerization.

### Analysis of *Cis*-acting elements in *FveCesA*/*Csl* promoters of strawberry Cellulose synthesis genes

**Figure 4:**
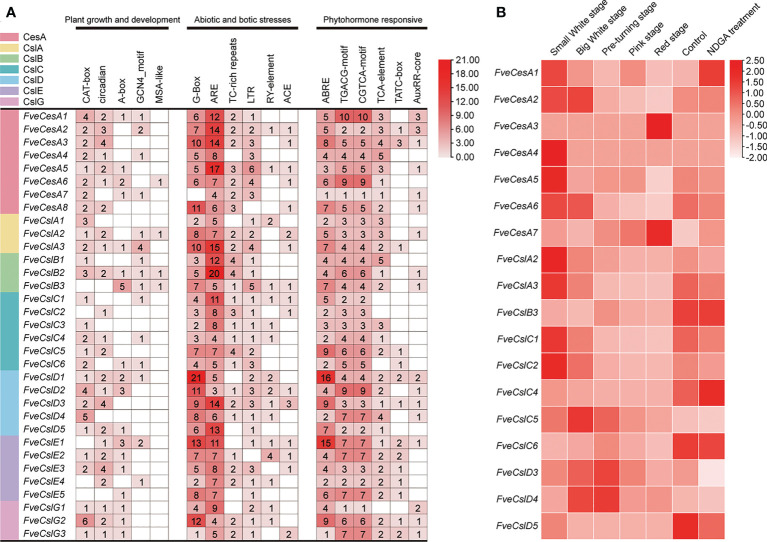
Promoter *cis*-acting elements and expression pattern analysis of *FveCesA/Csl* in strawberry. **(A)** Promoter *cis*-acting elements analysis of *FveCesA/Csl* genes. *CesA/Csl* subfamilies are distinguished by different colors. Number and color depth are represented the number of corresponding *cis*-elements. **(B)** Transcriptome analysis reveals the expression level of *FveCesA/Csl* genes during strawberry fruit development.

The *cis*-acting elements distributed in the regulatory regions of a promoter principally determine the gene expression pattern. The *cis*-acting elements in the promoter of the identified *FveCesA/Csl* genes were predicted. In total, 18 types of *cis*-acting elements were detected in the promoter of the *FveCesA/Csl* genes (**Figure 4A** **)**. These elements included a light-responsive element (G-box), defense and stress response elements (TC-rich-repeats, LTR-element, and ARE-element), phytohormone response elements (ABRE, TGACG-motif, CGTCA-motif, and TCA-element), and growth and development-related elements (circadian, CAT-box, and GCNA4-motif). In particular, the G-box, ARE-element, TGACG-motif, and CGTCA-motif were abundant in the promoters of most *FveCesA/Csl* genes, indicating that these genes were required for stress tolerance and the growth and development of strawberry, and were extensively regulated by abscisic acid (ABA) and jasmonic acid (JA) hormone signaling.

### Expression profiles of Cellulose synthesis genes during strawberry fruit ripening

To investigate the expression profiles of *FveCesA* and *FveCsl* genes during fruit development, we analyzed RNA-seq data for diploid woodland strawberry fruit at different developmental stages. The expression profiles of *FveCesA* and *FveCsl* genes in the transcriptome data were represented as a heat map (**Figure 4B**). The transcript levels of the *FveCesA1*, *2*, *4*, *5*, and *6*, *FveCslA2* and *3*, and *FveCslC1*, *2*, and *5* genes were significantly decreased in the late stage of fruit development compared with the early stage. Interestingly, after ABA biosynthesis was inhibited by nordihydroguaiaretic acid (NDGA) treatment, the expression levels of *FveCesA1* and *FveCslC4* were remarkably increased, whereas the expression levels of *FveCesA5* and *6*, *FveCslA2* and *3*, *FveCslC1* and *2*, and *FveCslD3* were significantly decreased. Therefore, the expression of these genes may be regulated by ABA signaling, which is required for strawberry fruit ripening.

**Figure 5:**
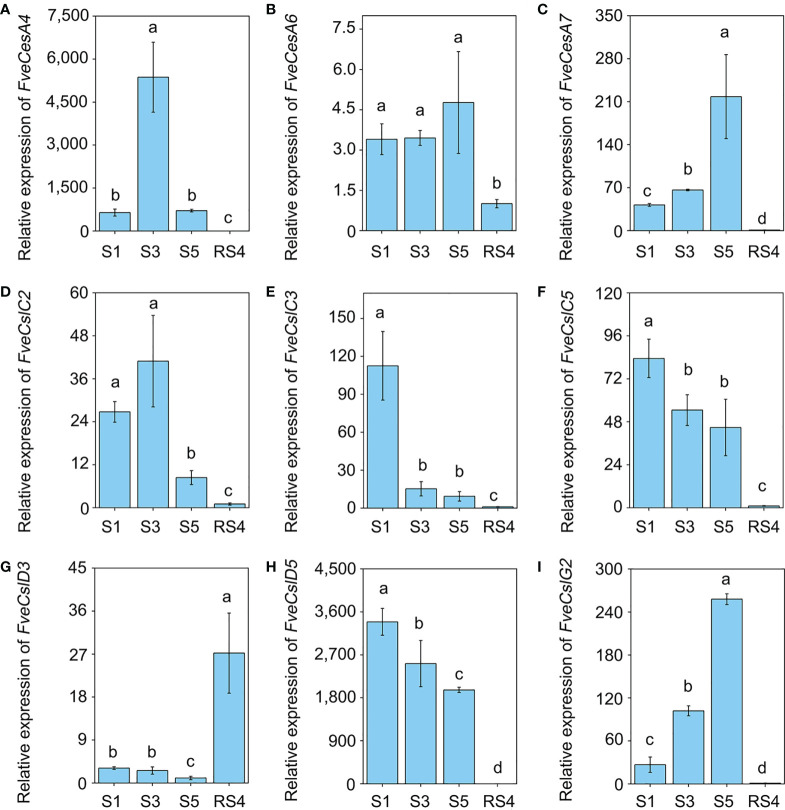
The expression pattern of *Cellulose synthase* genes during fruit development. **(A–I)** The relative expression levels of *FveCesA4* **(A)**, *FveCesA6* **(B)**, *FveCesA7* **(C)**, *FveCslC2* **(D)**, *FveCslC3* **(E)**, *FveCsl5* **(F)**, *FveCslD3* **(G)**, *FveCslD5* **(H)**, and *FveCslG2* **(I)** during fruit development were detected by qRT-PCR. Error bars represents SD of three independent replicates (5-10 fruits were used for each replicate). Letter in figure indicates significant differences between stages (*P* < 0.05, one-way ANOVA, Tukey’s HSD *post hoc* test).

To further verify the RNA-seq results, we divided diploid woodland strawberry fruit development, starting from anthesis, into 11 stages as described previously (Liao et al., 2018), comprising seven early stages (S1-S7) and four ripening stage (RS1-RS4). Based on the promoter and RNA-seq data, the transcript levels of 9 *FveCesA*/*Csl* genes were detected by qRT-PCR during the early and ripening stages of fruit development (**Figure 5**). In the early stages (S1, S3 and S5), the expression of *FveCesA4*, *FveCesA7* and *FveCslG2* was dramatically increased, but the expression of *FveCesA6* was not changed significantly. In the ripening stage (RS4), the expression of the four genes was significantly decreased (**Figures 5A–C, I**). With regard to *FveCslC2*, *FveCslC3*, *FveCslC5* and *FveCslD5*, the expression levels of were high at the S1 stage, but dramatically decreased with progression of fruit development (**Figures 5D–F, H**). Interestingly, the expression levels of *FveCslD3* remained low in the early stages, but showed a marked upward trend in the ripening stages (**Figure 5G**), suggesting that *FveCslD3* may have different function in the regulation of fruit development. These results indicated that *FveCesA* and *FveCsl* genes displayed functional diversity during fruit development.

### *FveCesA4* negatively regulates strawberry fruit ripening by enhancing fruit firmness

**Figure 6:**
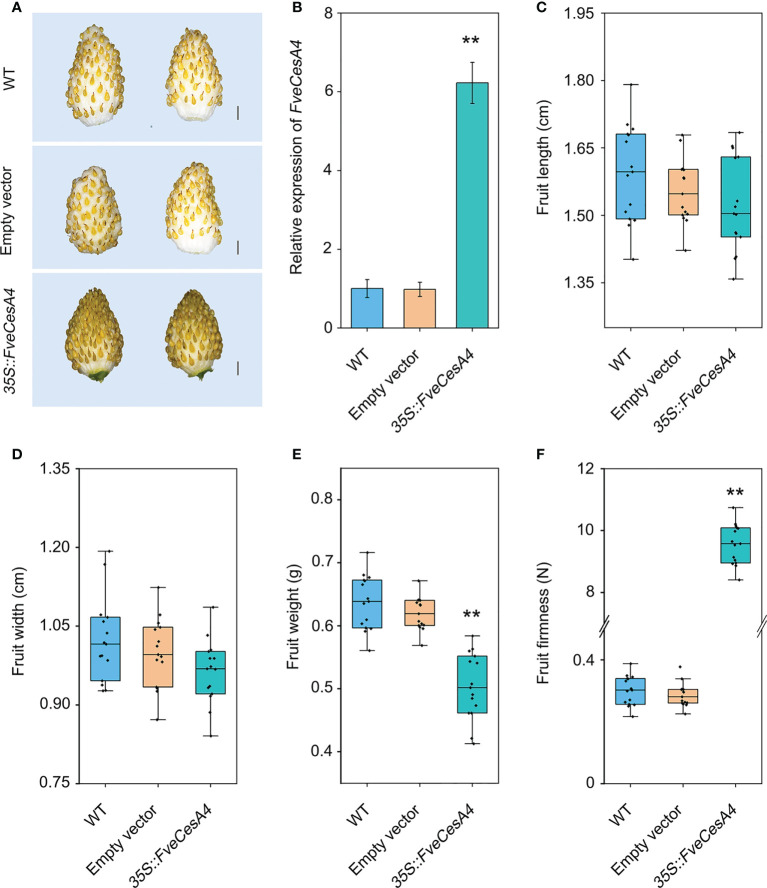
Transient overexpression of *FveCesA4* suppressed fruit ripening. *FveCesA4* was transiently overexpressed at S7 stage, and all analyses were performed at 7 days after infiltration. **(A)** Fruit ripening was delayed after overexpression of *FveCesA4*, whereas the control fruits ripened. Scale bar: 10 mm. **(B)** *FveCesA4* transcript levels in *35S::FveCesA4* fruits dramatically increased compared with control. **(C–F)** Fruit length **(C)**, width **(D)**, weight **(E)** and firmness **(F)** analyses of *35S::FveCesA4* fruits. All analyses were performed in three independent replicates with 15 fruits per replicate. ** indicates significant differences between groups (*P* < 0.05, one-way ANOVA, Tukey’s HSD *post hoc* test).

**Figure 7:**
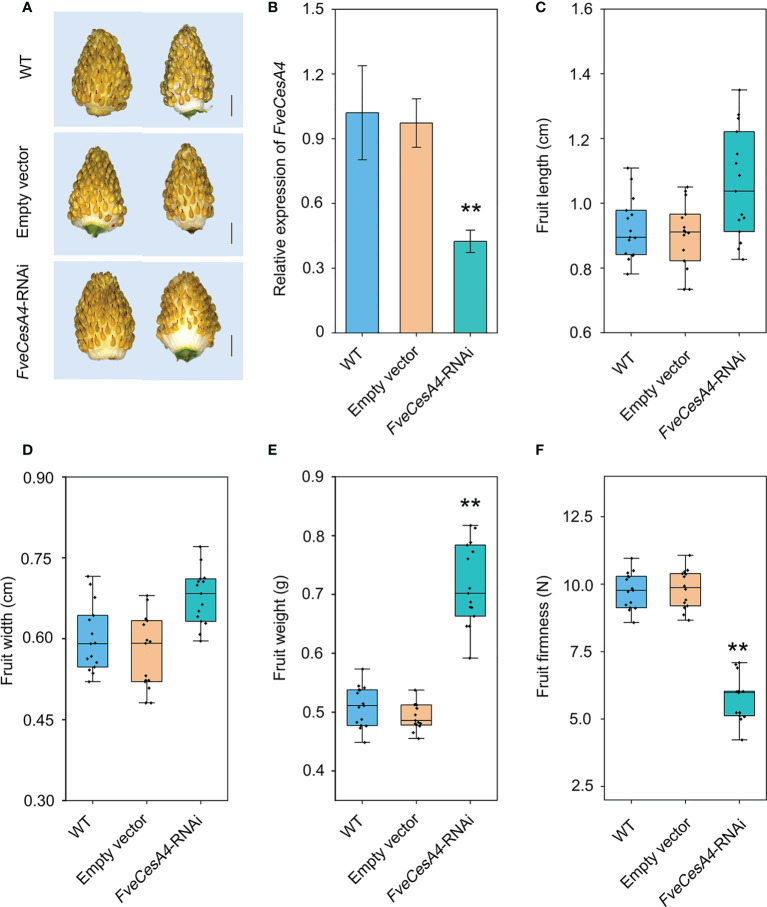
Transient silencing of *FveCesA4* accelerated fruit ripening. *FveCesA4* was transiently silenced at S4 stage, and all analyses were performed at 7 days after infiltration. **(A)** *FveCesA4-RNAi* fruits became ripened, while the control fruits remained unripe. Scale bar: 10 mm. **(B)** *FveCesA4* transcript levels in *FveCesA4-RNAi* fruits dramatically suppressed compared with control. **(C–F)** Fruit length **(C)**, width **(D)**, weight **(E)** and firmness **(F)** analyses of *FveCesA4-RNAi* fruits. All analyses were performed in three independent replicates with 15 fruits per replicate. ** indicates significant differences between groups (*P* < 0.05, one-way ANOVA, Tukey’s HSD *post hoc* test).

To investigate the function of cellulose synthesis genes in strawberry fruit ripening, *FveCesA4* was transiently overexpressed in the fruit at the S7 stage (**Figure 6A**). The expression level of *FveCesA4* in *35S::FveCesA4* fruit increased dramatically after injection (**Figure 6B**), resulting in reduced fruit weight and significantly enhanced fruit firmness compared with the mock control (**Figures 6E, F**). Next, we deployed RNAi of *FveCesA4* at the S4 stage using the VIGS system. The expression level of *FveCesA4* was greatly suppressed at 7 days after transient silencing (**Figures 7A, B**). Accompanying the suppression of *FveCesA* expression, significantly increased fruit weight and decreased fruit firmness in *FveCesA4-RNAi* fruit were observed, in comparison with those of the control (**Figures 7E, F**). In contrast, the fruit size and shape showed no significant change in *FveCesA4-RNAi* and *35S::FveCesA4* fruits (**Figures 6C, D**, **7C, D**). Taken together, these results indicated that FveCesA4 affects fruit ripening and biomass accumulation by regulating fruit firmness.

## Discussion

The *CesA* and *Csl* gene families associated with cell wall polysaccharide biosynthesis play important roles in plant growth and development and responses to environmental stress (McFarlane et al., 2014). To date, the *CesA/Csl* gene families have been extensively identified and studied in various species, such as *Arabidopsis* (Persson et al., 2007), rice (Hazen et al., 2002), cotton (Pear et al., 1996) and banana (Yuan et al., 2021), but they have not been reported previously in strawberry. In this study, eight *FveCesA* genes and 25 *FveCsl* genes were identified in diploid woodland strawberry by genome-wide sequence analysis. Further analyses of phylogenic relationships, *cis*-acting elements in promoter, synteny, gene expression patterns, subcellular localization, and transient transformation provide clues for understanding the mechanism of CesA/Csl function in the development and ripening of a non-climacteric fruit.

Previous studies have demonstrated that the cellulose synthase gene superfamily can be resolved into the CesA clade and ten Csl clades, (i.e., CslA-CslH, CslJ and CslM) (McFarlane et al., 2014). Among the Csl clades, the CslA, CslC and CslD lineages are prevalent in all terrestrial plant (Farrokhi et al., 2006). The CslB lineages is considered to be dicotyledons-specific, whereas the CslF and CslH lineages have been indicated to be restricted to grasses (Yin et al., 2009). The present phylogenetic analysis showed that the 33 putative FveCesA/Csl proteins were dividable into seven distinct subfamilies (**Figure 1**). Eight putative proteins were clustered into the CesA clade, as the largest clade, and the remaining putative proteins were grouped into the CslA, B, C, D, E, and G clades with different abundances, suggesting that the *CesA*/*Csl* gene family members have undergone extensive expansion and diversification during evolution. The CslD clade was phylogenetically close to the CesA clade, indicating that these two lineages shared a common ancestor. No *FveCesA/Csl* genes were resolved in the CslF and CslH clades, which was consistent with previous reports (Yin et al., 2009). The CslA and CslC clades are considered to have a distinct evolutionary origins from the remaining Csl clades due to their close relationship with the single-copy homologue found in six green algae species (Yin et al., 2009). The present analysis of structural characteristics demonstrated that the CslA and CslC clades in strawberry contained the glycosyl transferase family 2 domain, whereas the remaining clades contained the cellulose synthase domain (**Figure 3C**). Similar to the conserved domain analysis, the motif components of strawberry *CesA*, *CslB*, *CslD*, *CslE* and *CslG* genes exhibited high similarity, whereas the *CslA* and *CslC* genes had entirely distinct motif compositions (**Figure 3B**). These results were consistent with those of previous reports in the other species. Furthermore, the *CslE* genes were distributed in tandem in strawberry Fvb3 chromosome (**Figure 2A**). It is speculated that *FveCslE* genes may be significantly expanded in strawberry genome possibly through tandem gene replication, which is similar to the conclusions of a study (Yin et al., 2009).

*CesA* and *Csl* family genes encode GT2-tyoe glycosyltransferases, which is characterized by conserved cytosolic substrate binding and catalytic residues composed of D, D, D, and QxxRW motifs (McFarlane et al., 2014). The first two D residues coordinate UDP, the third D residue promotes the glucan extension, and the QxxRW residues are responsible for binding the glycogen residue at the terminus of cellulose chain (Morgan et al., 2013). The present amino acid sequence alignment showed that almost all FveCesA/Csl proteins typically contained D, D, D, and QxxRW motifs (**Supplementary Figures S1**–**S4**). The substitution of glutamine residues in the QxxRW motif of FveCslE1, FveCslE2, and FveCslG2 proteins by other residues implies that they may have different functional properties (**Supplementary Figure S3**), further supporting the hypothesis that the CslE and CslG clade genes have acquired new plant-specific functions through diversification during evolution (Yin et al., 2009). Cellulose synthesis in plants is catalyzed by the rosette-like cellulose synthesis complex localized on the plasma membrane (McFarlane et al., 2014). The present prediction of subcellular localization showed that all the FveCesA/Csl proteins were localized on the membrane (**Supplementary Table S2**). In addition, proteins in the CesA and Csl clades contained the conserved N-terminal zinc-finger domain (**Supplementary Figures S1**, **S2**), which may be involved in CesA functions specific to higher plants, such as the formation of rosette structure through subunit multimerization and the interaction with regulatory factors (McFarlane et al., 2014).

In general, strawberry fruit firmness and texture change dramatically during development and are regulated by ABA signaling (Liao et al., 2018). Endogenous ABA levels in strawberry fruit remain extremely low level during the early fruit development, but increases sharply in the fruit ripening stage, which, in turn triggers fruit ripening events, such as fruit softening and soluble sugars accumulation (Liao et al., 2018). Fruit firmness and texture are closely related to the composition and content of cell wall catalyzed mainly by cellulose synthesis genes. In the present study, we analyzed the promoters of the *FveCesA*/*Csl* superfamily members (**Figure 4A**). Abundant *cis*-acting elements associated with plant growth and development, stress response, and hormone regulation were detected in the promoters of the *FveCesA*/*Csl* genes. These results suggested that the *FveCesA*/*Csl* gene family may participate in plant growth and development as well as stress tolerance, and the family members are regulated by plant hormones, especially ABA signaling. Consistent with the present qRT-PCR results (**Figure 5**), the transcriptome data showed that the expression levels of several *FveCesA*/*Csl* genes decreased to varying extents in the fruit ripening stage (**Figure 4B**). Furthermore, after spray application of NDGA to reduce the endogenous ABA content in fruit, the transcript abundance of certain *FveCesA/Csl* genes was changed to some extent (**Figure 4B**). Moreover, the strawberry fruit transiently overexpressing *FveCesA4* resulted in significantly increased fruit texture firmness and delayed fruit ripening (**Figures 6A, B, F**). The fruit transient silencing of *FveCesA4* in strawberry showed a similar conclusion (**Figures 7A, B, F**), implying that *FveCesA4* positively regulates fruit firmness and inhibits fruit ripening. In rice, overexpression of *OsCslD4* increases endogenous ABA levels and enhances rice salt-tolerance (Zhao et al., 2022). Whether the transient overexpression of *FveCesA4* in strawberry fruit also affects endogenous ABA abundance, which is required for fruit ripening, through the alteration of cell wall integrity remains to be determined, and the underling mechanism needs to be further examined. Notably, previous reports have shown that cellulose synthesis genes not only function in plant growth and development but also enhance plant biomass (Hu et al., 2018a). Overexpression of *AtCesA2*, *AtCesA5* and *AtCesA6* enhances secondary cell wall deposition and results in increased biomass production in *Arabidopsis* (Hu et al., 2018a). However, the present data showed that overexpression of *FveCesA4* in strawberry fruit contributed to reduced fruit biomass (**Figure 6E**). The RNAi results indicated a similar conclusion (**Figure 7E**). One possible explanation could be that overexpression of *FveCesA4* contributed to improved fruit firmness and inhibited fruit ripening, which, in turn suppressed the accumulation of relevant metabolites and then reduced fruit biomass. Auxin and gibberellin (GA) promote strawberry fruit growth in the early phase. The contents of endogenous IAA and GA_1+3_ increased to the highest at S3 stage during fruit development (Liao et al., 2018). In this study, *FveCesA4* exhibited the highest expression level at S3 stage (**Figure 5A**), and was phylogenetically closed to *AtCesA4* (**Figure 1**), which was responsible for the synthesis of secondary cell wall cellulose, suggesting that *FveCesA4*- mediated cell wall synthesis may be regulated by auxin and GA signaling and play an important role in regulating fruit early development. Hence, the underlying mechanism of *FveCesA/Csl*-mediated cell wall synthesis in the regulation of strawberry fruit development and ripening requires further exploration.

## Conclusion

In this study, we identified eight *FveCesA* genes and 25 *FveCsl* genes in the genome of diploid woodland strawberry. Analyses of the protein structure, phylogenetic relationships, and expression patterns were performed to investigate the characteristics and functions of the *FveCesA*/*Csl* genes. The transient transformation of *FveCesA4* in fruit further confirmed that cell wall synthesis is essential for strawberry fruit development and ripening. The present results provide a foundation for detailed explorations of whether *FveCesA/Csl*-mediated cell wall synthesis regulates fruit ripening in strawberry, and they will assist in strawberry breeding aimed at improving of the shelf-life of strawberry fruits.

## Data availability statement

The original contributions presented in the study are included in the article/**Supplementary Material**. Further inquiries can be directed to the corresponding author.

## Author contributions

HH, SZ, and XL designed the research, analyzed the data, and revised the final manuscript. HH and SZ performed the most of the experiments, with assistance from MX, SL, JC, TL, GG, RW, JL, and YS. SZ and XL wrote the manuscript. All authors contributed to the article and approved the submitted version.

## Funding

This study was funded by the National Natural Science Foundation of China (No. 31801840).

## Conflict of interest

The authors declare that the research was conducted in the absence of any commercial or financial relationships that could be construed as a potential conflict of interest.

## Publisher’s note

All claims expressed in this article are solely those of the authors and do not necessarily represent those of their affiliated organizations, or those of the publisher, the editors and the reviewers. Any product that may be evaluated in this article, or claim that may be made by its manufacturer, is not guaranteed or endorsed by the publisher.

## Supplementary material

Supplementary Figure 1 Amino acid sequence alignment of CesA subfamily in strawberry. Conserved zinc-binding domain is highlighted by red line. The conserved cysteine residues in zinc-binding domain are marked with red stars. Conserved cellulose_synt domain is highlighted by blue line. The conserved “D, D, D and QXXRW motif” are marked with blue stars.

Supplementary Figure 2 Amino acid sequence alignment of CslD subfamily in strawberry. Conserved zinc-binding domain is highlighted by red line. The conserved cysteine residues in zinc-binding domain are marked with red stars. Conserved cellulose_synt domain is highlighted by blue line. The conserved “D, D, D and QXXRW motif” are marked with blue stars.

Supplementary Figure 3 Amino acid sequence alignment of CslA and CslC subfamily in strawberry. **(A)** Amino acid sequence alignment of FveCslAs proteins. **(B)** Amino acid sequence alignment of FveCslCs proteins. Conserved Glyco_tranf_2_3 domain is highlighted by blue line. The conserved “D, D, D and QXXRW motif” are marked with blue stars.

Supplementary Figure 4 Amino acid sequence alignment of CslB, CslE and CslG subfamily in strawberry. **(A)** Amino acid sequence alignment of FveCslBs proteins. **(B)** Amino acid sequence alignment of FveCslEs proteins. **(C)** Amino acid sequence alignment of FveCslGs proteins. Conserved Glyco_tranf_2_3 domain is highlighted by blue line. The conserved “D, D, D and QXXRW motif” are marked with blue stars.

## References

[B1] Appenzeller L. Doblin M. Barreiro R. Wang H. Niu X. Kollipara K.. (2004). Cellulose synthesis in maize: isolation and expression analysis of the cellulose synthase (CesA) gene family. Cellulose 11 (3), 287–299. doi: 10.1023/B:CELL.0000046417.84715.27

[B2] Bernal A. J. Jensen J. K. Harholt J. Sørensen S. Moller I. Blaukopf C.. (2007). Disruption of ATCSLD5 results in reduced growth, reduced xylan and homogalacturonan synthase activity and altered xylan occurrence in arabidopsis. Plant J. 52 (5), 791–802. doi: 10.1111/j.1365-313X.2007.03281.x 17892446

[B3] Cantarel B. L. Coutinho P. M. Rancurel C. Bernard T. Lombard V. Henrissat B. (2009). The carbohydrate-active EnZymes database (CAZy): an expert resource for glycogenomics. Nucleic Acids Res. 37 (suppl_1), D233–D238. doi: 10.1093/nar/gkn663 18838391 PMC2686590

[B4] Chen C. Chen H. Zhang Y. Thomas H. R. Frank M. H. He Y.. (2020). TBtools: an integrative toolkit developed for interactive analyses of big biological data. Mol. Plant 13 (8), 1194–1202. doi: 10.1016/j.molp.2020.06.009 32585190

[B5] Choe S. Choi B. Kang J. H. Seo J. K. (2021). Tolerance to tomato yellow leaf curl virus in transgenic tomato overexpressing a cellulose synthase-like gene. Plant Biotechnol. J. 19 (4), 657. doi: 10.1111/pbi.13539 33378588 PMC8051598

[B6] Cosgrove D. J. (2005). Growth of the plant cell wall. Nat. Rev. Mol. Cell Biol. 6 (11), 850–861. doi: 10.1038/nrm1746 16261190

[B7] Czaja W. Krystynowicz A. Kawecki M. Wysota K. Sakiel S. Wróblewski P.. (2007). Cellulose: Molecular and structural biology (The Netherlands: Springer Dordrecht). doi: 10.1007/978-1-4020-5380-1

[B8] Desprez T. Juraniec M. Crowell E. F. Jouy H. Pochylova Z. Parcy F.. (2007). Organization of cellulose synthase complexes involved in primary cell wall synthesis in arabidopsis thaliana. Proc. Natl. Acad. Sci. U.S.A. 104 (39), 15572–15577. doi: 10.1073/pnas.0706569104 17878303 PMC2000492

[B9] Dhugga K. S. (2012). Biosynthesis of non-cellulosic polysaccharides of plant cell walls. Phytochemistry 74, 8–19. doi: 10.1016/j.phytochem.2011.10.003 22137036

[B10] Dhugga K. S. Barreiro R. Whitten B. Stecca K. Hazebroek J. Randhawa G. S.. (2004). Guar seed ß-mannan synthase is a member of the cellulose synthase super gene family. Science 303 (5656), 363–366. doi: 10.1126/science.1090908 14726589

[B11] Farrokhi N. Burton R. A. Brownfield L. Hrmova M. Wilson S. M. Bacic A.. (2006). Plant cell wall biosynthesis: genetic, biochemical and functional genomics approaches to the identification of key genes. Plant Biotechnol. J. 4 (2), 145–167. doi: 10.1111/j.1467-7652.2005.00169.x 17177793

[B12] Goubet F. Barton C. J. Mortimer J. C. Yu X. Zhang Z. Miles G. P.. (2009). Cell wall glucomannan in arabidopsis is synthesised by CSLA glycosyltransferases, and influences the progression of embryogenesis. Plant J. 60 (3), 527–538. doi: 10.1111/j.1365-313x.2009.03977.x 19619156

[B13] Gu T. Jia S. Huang X. Wang L. Fu W. Huo G.. (2019). Transcriptome and hormone analyses provide insights into hormonal regulation in strawberry ripening. Planta 250 (1), 145–162. doi: 10.1007/s00425-019-03155-w 30949762

[B14] Hazen S. P. Scott-Craig J. S. Walton J. D. (2002). Cellulose synthase-like genes of rice. Plant Physiol. 128 (2), 336–340. doi: 10.1104/pp.010875 11842136 PMC1540205

[B15] Hu H. Zhang R. Feng S. Wang Y. Wang Y. Fan C.. (2018a). Three AtCesA6-like members enhance biomass production by distinctively promoting cell growth in arabidopsis. Plant Biotechnol. J. 16 (5), 976–988. doi: 10.1111/j.1467-7652.2005.00169.x 28944540 PMC5902768

[B16] Hu H. Zhang R. Tao Z. Li X. Li Y. Huang J.. (2018b). Cellulose synthase mutants distinctively affect cell growth and cell wall integrity for plant biomass production in arabidopsis. Plant Cell Physiol. 59 (6), 1144–1157. doi: 10.1093/pcp/pcy050 29514326

[B17] Kumar S. Stecher G. Tamura K. (2016). MEGA7: molecular evolutionary genetics analysis version 7.0 for bigger datasets. Mol. Biol. Evol. 33 (7), 1870–1874. doi: 10.1093/molbev/msw054 27004904 PMC8210823

[B18] Liao X. Li M. Liu B. Yan M. Yu X. Zi H.. (2018). Interlinked regulatory loops of ABA catabolism and biosynthesis coordinate fruit growth and ripening in woodland strawberry. Proc. Natl. Acad. Sci. U.S.A. 115 (49), E11542–E11550. doi: 10.1073/pnas.1812575115 30455308 PMC6298082

[B19] Li Y. Cheng X. Fu Y. Wu Q. Guo Y. Peng J.. (2019). A genome-wide analysis of the cellulose synthase-like (Csl) gene family in maize. Biol. Plant 63 (1), 721–732. doi: 10.1186/s12870-017-1142-z

[B20] Liepman A. H. Cavalier D. M. (2012). The cellulose synthase-like a and cellulose synthase-like c families: recent advances and future perspectives. Front. Plant Sci. 3. doi: 10.3389/fpls.2012.00109 PMC3359485 22654891

[B21] Liepman A. H. Wilkerson C. G. Keegstra K. (2005). Expression of cellulose synthase-like (Csl) genes in insect cells reveals that CslA family members encode mannan synthases. Proc. Natl. Acad. Sci. U.S.A. 102 (6), 2221–2226. doi: 10.1073/pnas.0409179102 15647349 PMC548565

[B22] Little A. Schwerdt J. G. Shirley N. J. Khor S. F. Neumann K. O’Donovan L. A.. (2018). Revised phylogeny of the cellulose synthase gene superfamily: insights into cell wall evolution. Plant Physiol. 177 (3), 1124–1141. doi: 10.1104/pp.17.01718 29780036 PMC6052982

[B23] Liu Z. Schneider R. Kesten C. Zhang Y. Somssich M. Zhang Y.. (2016). Cellulose-microtubule uncoupling proteins prevent lateral displacement of microtubules during cellulose synthesis in arabidopsis. Dev. Cell 38 (3), 305–315. doi: 10.1016/j.devcel.2016.06.032 27477947

[B24] Malinovsky F. G. Fangel J. U. Willats W. G. (2014). The role of the cell wall in plant immunity. Front. Plant Sci. 5. doi: 10.3389/fpls.2014.00178 PMC4018530 24834069

[B25] McFarlane H. E. Döring A. Persson S. (2014). The cell biology of cellulose synthesis. Annu. Rev. Plant Biol. 65 (1), 69–94. doi: 10.1146/annurev-arplant-050213-040240 24579997

[B26] Morgan J. L. Strumillo J. Zimmer J. (2013). Crystallographic snapshot of cellulose synthesis and membrane translocation. Nature 493 (7431), 181–186. doi: 10.1038/nature11744 23222542 PMC3542415

[B27] Moya-León M. A. Mattus-Araya E. Herrera R. (2019). Molecular events occurring during softening of strawberry fruit. Front. Plant Sci. 10. doi: 10.3389/fpls.2019.00615 PMC6529986 31156678

[B28] Park S. Szumlanski A. L. Gu F. Guo F. Nielsen E. (2011). A role for CSLD3 during cell-wall synthesis in apical plasma membranes of tip-growing root-hair cells. Nat. Cell Biol. 13 (8), 973–980. doi: 10.1038/ncb2294 21765420

[B29] Pear J. R. Kawagoe Y. Schreckengost W. E. Delmer D. P. Stalker D. M. (1996). Higher plants contain homologs of the bacterial celA genes encoding the catalytic subunit of cellulose synthase. Proc. Natl. Acad. Sci. U.S.A. 93 (22), 12637–12642. doi: 10.1073/pnas.93.22.12637 8901635 PMC38045

[B30] Persson S. Paredez A. Carroll A. Palsdottir H. Doblin M. Poindexter P.. (2007). Genetic evidence for three unique components in primary cell-wall cellulose synthase complexes in arabidopsis. Proc. Natl. Acad. Sci. U.S.A. 104 (39), 15566–15571. doi: 10.1073/pnas.0706592104 17878302 PMC2000526

[B31] Richmond T. A. Somerville C. R. (2000). The cellulose synthase superfamily. Plant Physiol. 124 (2), 495–498. doi: 10.1104/pp.124.2.495 11027699 PMC1539280

[B32] Roberts A. W. Bushoven J. T. (2007). The cellulose synthase (CESA) gene superfamily of the moss physcomitrella patens. Plant Mol. Biol. 63 (2), 207–219. doi: 10.1007/s11103-006-9083-1 17006591

[B33] Song X. Xu L. Yu J. Tian P. Hu X. Wang Q.. (2019). Genome-wide characterization of the cellulose synthase gene superfamily in solanum lycopersicum. Gene 688, 71–83. doi: 10.1016/j.gene.2018.11.039 30453073

[B34] Taylor N. G. Howells R. M. Huttly A. K. Vickers K. Turner S. R. (2003). Interactions among three distinct CesA proteins essential for cellulose synthesis. Proc. Natl. Acad. Sci. U.S.A. 100 (3), 1450–1455. doi: 10.1073/pnas.0337628100 12538856 PMC298793

[B35] Verhertbruggen Y. Yin L. Oikawa A. Scheller H. V. (2011). Mannan synthase activity in the CSLD family. Plant Signal. Behav. 6 (10), 1620–1623. doi: 10.4161/psb.6.10.17989 21904114 PMC3256401

[B36] Wang Q. Wang M. Chen J. Qi W. Lai J. Ma Z.. (2022). ENB1 encodes a cellulose synthase 5 that directs synthesis of cell wall ingrowths in maize basal endosperm transfer cells. Plant Cell 34 (3), 1054–1074. doi: 10.1093/plcell/koab312 34935984 PMC8894971

[B37] Yang J. Bak G. Burgin T. Barnes W. J. Mayes H. B. Peña M. J.. (2020). Biochemical and genetic analysis identify CSLD3 as a beta-1, 4-glucan synthase that functions during plant cell wall synthesis. Plant Cell 32 (5), 1749–1767. doi: 10.1105/tpc.19.00637 32169960 PMC7203914

[B38] Yin Y. Huang J. Xu Y. (2009). The cellulose synthase superfamily in fully sequenced plants and algae. BMC Plant Biol. 9 (1), 1–14. doi: 10.1186/1471-2229-9-99 19646250 PMC3091534

[B39] Yin L. Verhertbruggen Y. Oikawa A. Manisseri C. Knierim B. Prak L.. (2011). The cooperative activities of CSLD2, CSLD3, and CSLD5 are required for normal arabidopsis development. Mol. Plant 4 (6), 1024–1037. doi: 10.1093/mp/ssr026 21471331

[B40] Yuan W. Liu J. Takáč T. Chen H. Li X. Meng J.. (2021). Genome-wide identification of banana csl gene family and their different responses to low temperature between chilling-sensitive and tolerant cultivars. Plants 10 (1), 122. doi: 10.3390/plants10010122 33435621 PMC7827608

[B41] Zhao H. Li Z. Wang Y. Wang J. Xiao M. Liu H.. (2022). Cellulose synthase-like protein OsCSLD4 plays an important role in the response of rice to salt stress by mediating abscisic acid biosynthesis to regulate osmotic stress tolerance. Plant Biotechnol. J. 20 (3), 468. doi: 10.1111/pbi.13729 34664356 PMC8882776

