# Supplementary figures and images for: Genome-wide identification and functional analysis of Cellulose synthase gene superfamily in Fragaria vesca
Source: Front Plant Sci. 2022 Nov 3;13:1044029. doi: 10.3389/fpls.2022.1044029 (PMC9669642; doi:10.3389/fpls.2022.1044029)

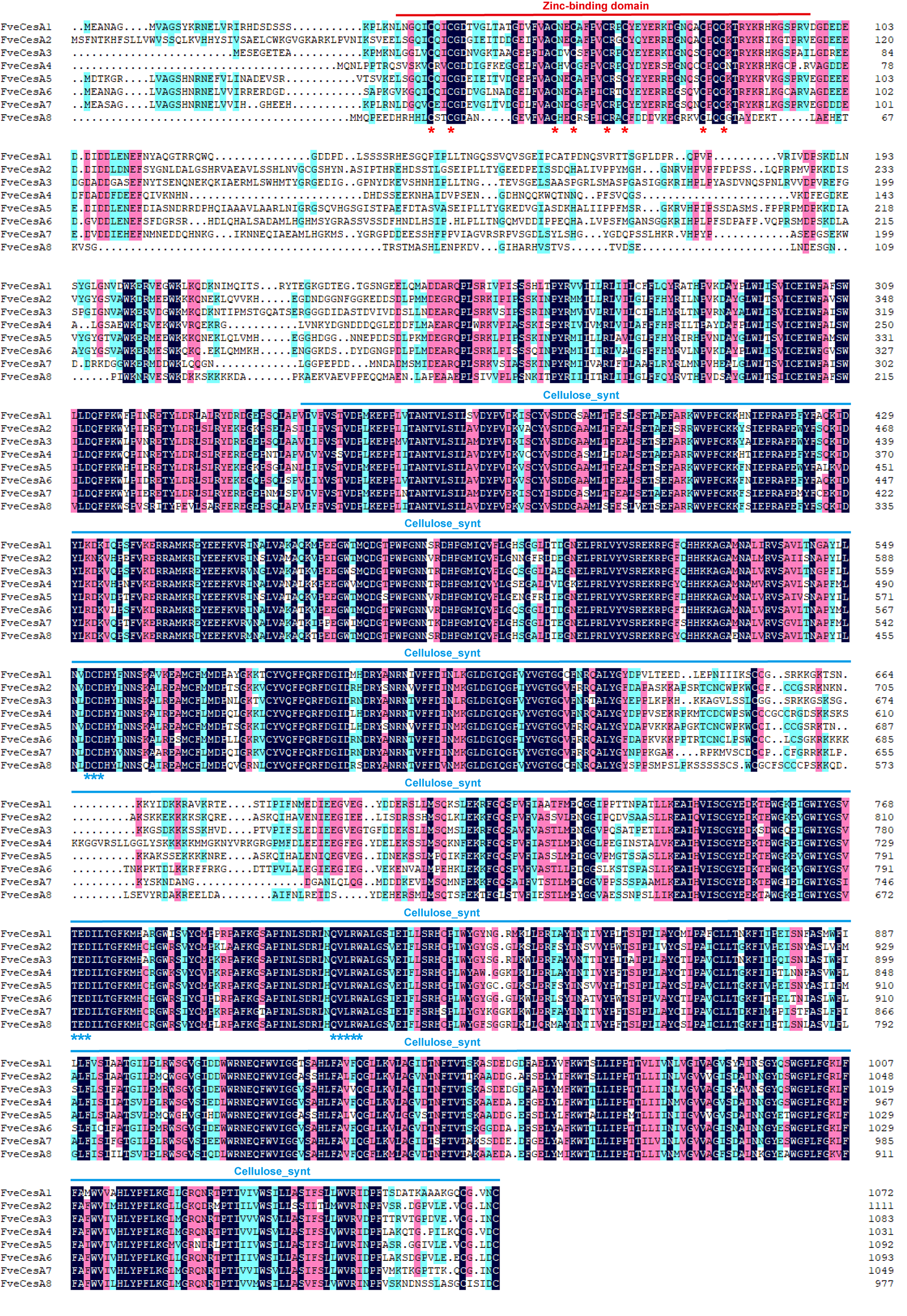

Supplement: Supplementary Figure 1 — Amino acid sequence alignment of CesA subfamily in strawberry. Conserved zinc-binding domain is highlighted by red line. The conserved cysteine residues in zinc-binding domain are marked with red stars. Conserved cellulose_synt domain is highlighted by blue line. The conserved “D, D, D and QXXRW motif” are marked with blue stars. [file Image_1.tif]

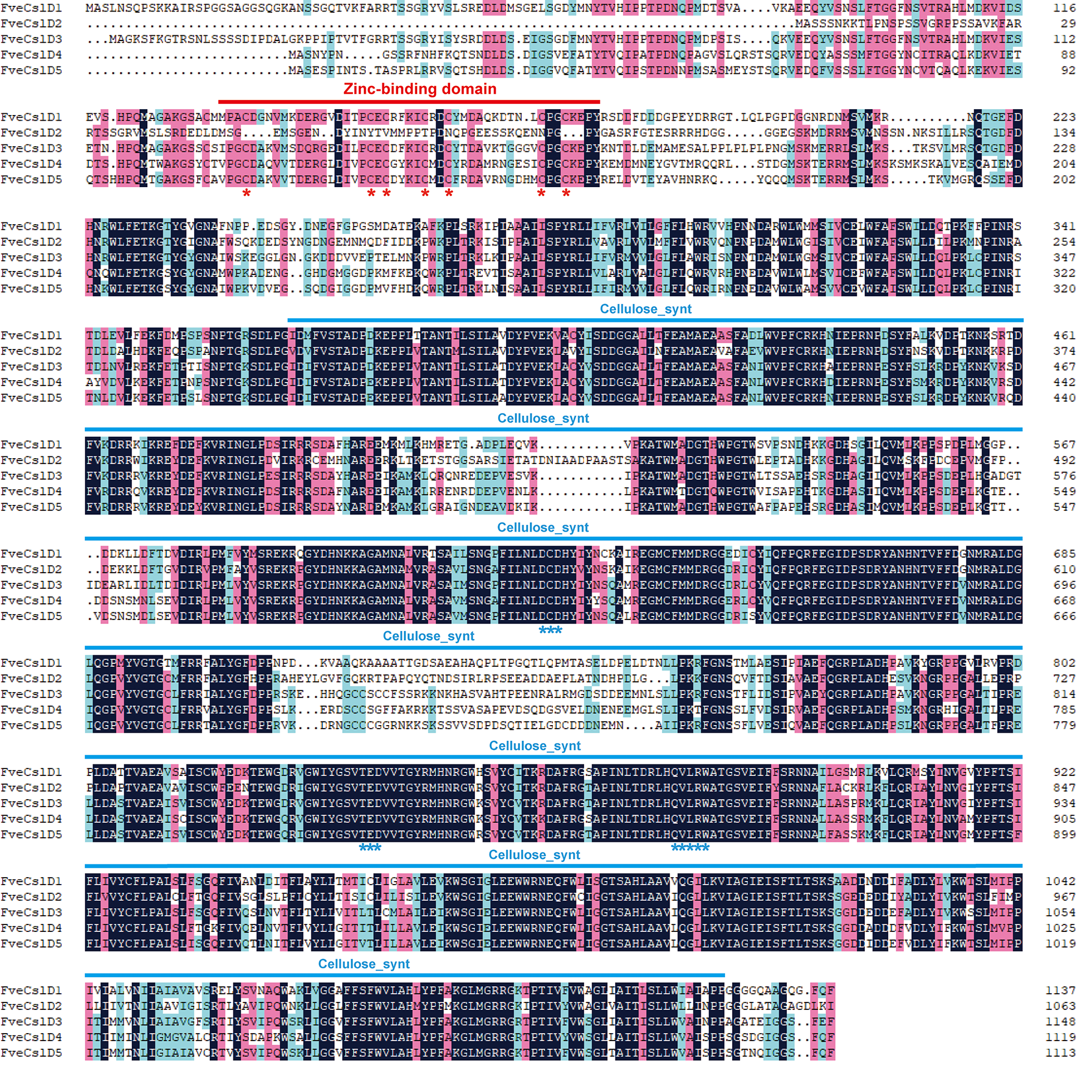

Supplement: Supplementary Figure 2 — Amino acid sequence alignment of CslD subfamily in strawberry. Conserved zinc-binding domain is highlighted by red line. The conserved cysteine residues in zinc-binding domain are marked with red stars. Conserved cellulose_synt domain is highlighted by blue line. The conserved “D, D, D and QXXRW motif” are marked with blue stars. [file Image_2.tif]

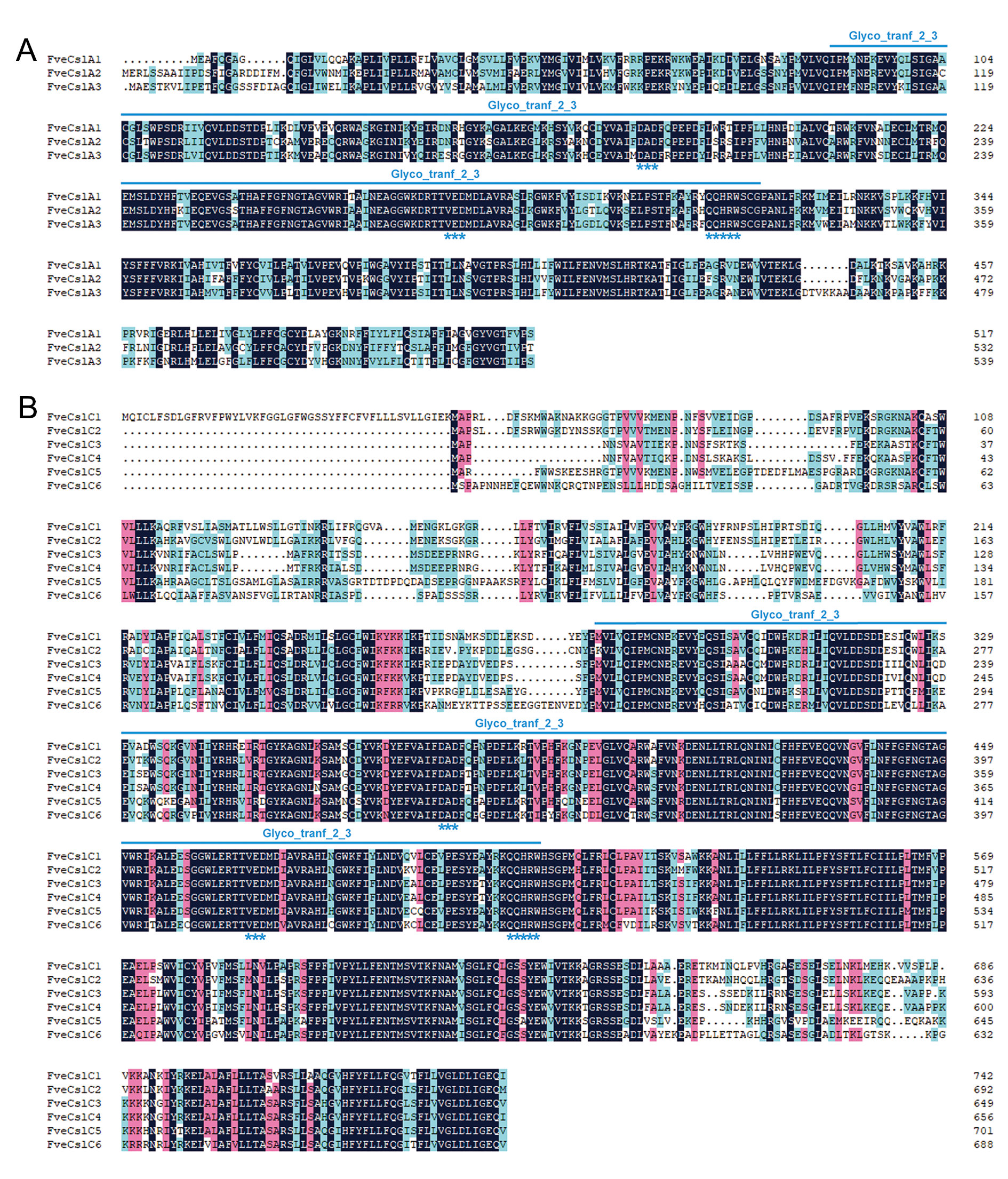

Supplement: Supplementary Figure 3 — Amino acid sequence alignment of CslA and CslC subfamily in strawberry. (A) Amino acid sequence alignment of FveCslAs proteins. (B) Amino acid sequence alignment of FveCslCs proteins. Conserved Glyco_tranf_2_3 domain is highlighted by blue line. The conserved “D, D, D and QXXRW motif” are marked with blue stars. [file Image_3.tif]

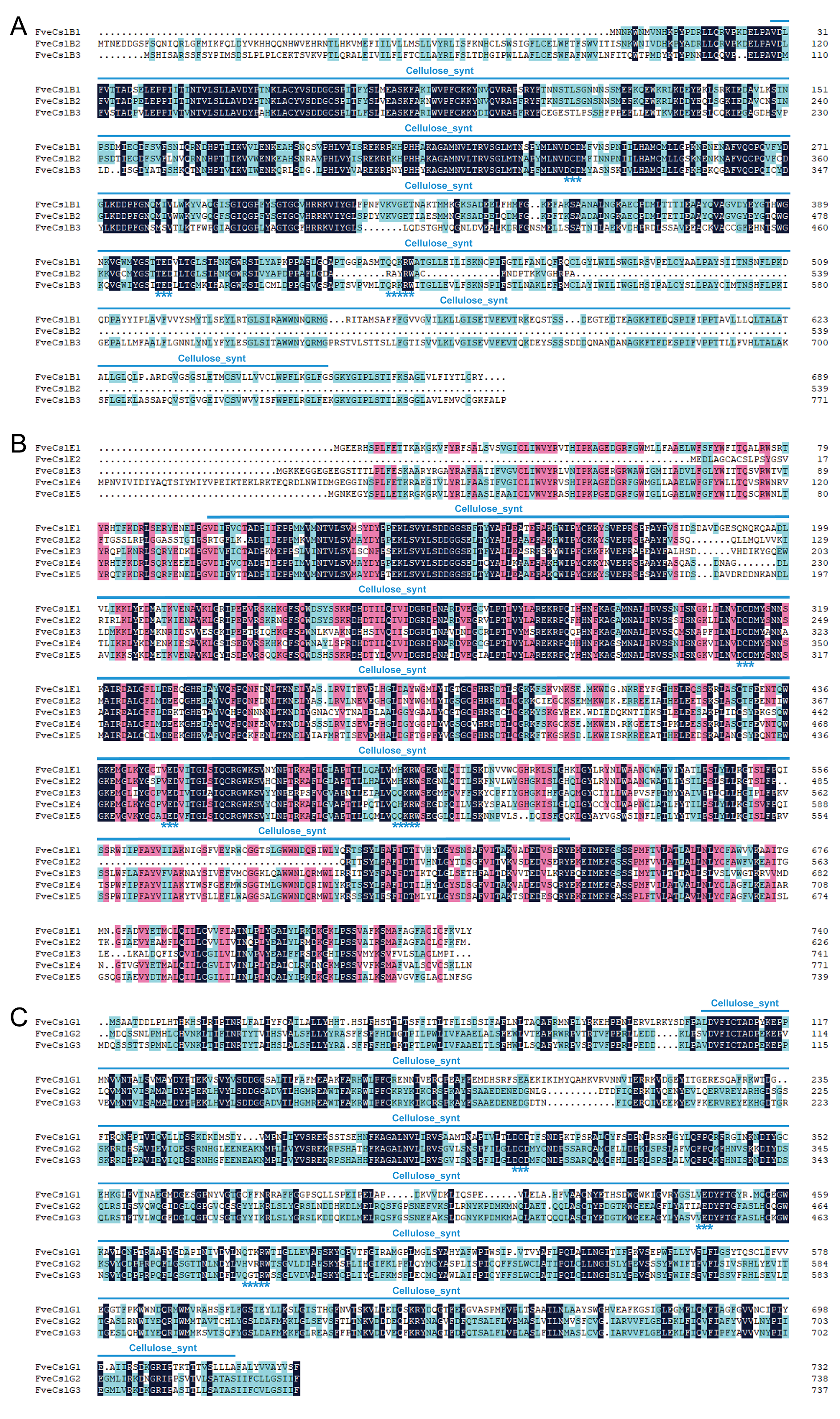

Supplement: Supplementary Figure 4 — Amino acid sequence alignment of CslB, CslE and CslG subfamily in strawberry. (A) Amino acid sequence alignment of FveCslBs proteins. (B) Amino acid sequence alignment of FveCslEs proteins. (C) Amino acid sequence alignment of FveCslGs proteins. Conserved Glyco_tranf_2_3 domain is highlighted by blue line. The conserved “D, D, D and QXXRW motif” are marked with blue stars. [file Image_4.tif]
